# Progenitor-Based Cell Biological Aspects of Neocortex Development and Evolution

**DOI:** 10.3389/fcell.2022.892922

**Published:** 2022-05-02

**Authors:** Samir Vaid, Wieland B. Huttner

**Affiliations:** ^1^ Department of Basic Neurosciences, University of Geneva, Geneva, Switzerland; ^2^ Max Planck Institute of Molecular Cell Biology and Genetics, Dresden, Germany

**Keywords:** basal process, apical process, centrosome, primary cilia, adherens junction, spindle orientation, delamination, cell cycle

## Abstract

During development, the decision of stem and progenitor cells to switch from proliferation to differentiation is of critical importance for the overall size of an organ. Too early a switch will deplete the stem/progenitor cell pool, and too late a switch will not generate the required differentiated cell types. With a focus on the developing neocortex, a six-layered structure constituting the major part of the cerebral cortex in mammals, we discuss here the cell biological features that are crucial to ensure the appropriate proliferation vs. differentiation decision in the neural progenitor cells. In the last two decades, the neural progenitor cells giving rise to the diverse types of neurons that function in the neocortex have been intensely investigated for their role in cortical expansion and gyrification. In this review, we will first describe these different progenitor types and their diversity. We will then review the various cell biological features associated with the cell fate decisions of these progenitor cells, with emphasis on the role of the radial processes emanating from these progenitor cells. We will also discuss the species-specific differences in these cell biological features that have allowed for the evolutionary expansion of the neocortex in humans. Finally, we will discuss the emerging role of cell cycle parameters in neocortical expansion.

## 1 Introduction

The neocortex is a six-layered neuronal structure that is part of the cerebral cortex of the brain. The neocortex is unique to mammals and is evolutionarily the newest part of the mammalian brain. Its importance lies in the facts that this part of the brain has expanded the most during human brain evolution and is associated with complex and higher order brain functions like cognitive abilities and language. Development of the neocortex is based on spatio-temporally restricted transcriptional programs that unfold in a sequential manner and are a predominant factor for the neural progenitor cell proliferation, differentiation, migration and specification of different neuronal subtypes in the neocortex (Telley et al., 2019; Vaid and Huttner, 2020; Ruan et al., 2021; Bandler et al., 2022). In addition, specific cell biological processes underlie the proper development of the mammalian neocortex and influence these transcriptional programs.

**FIGURE 1:**
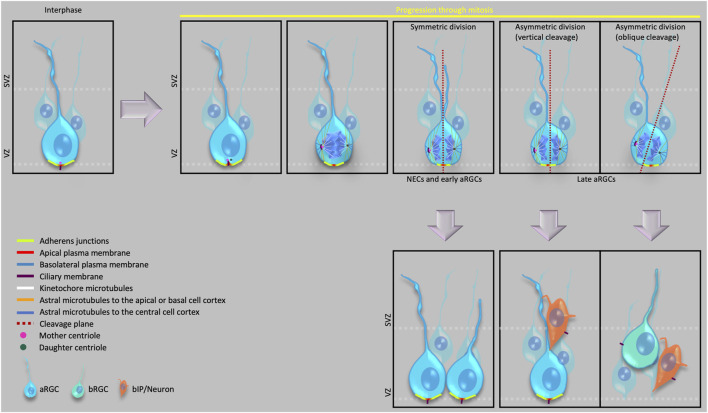
Cell biological features of apical progenitors and their various modes of division.

In recent years, advancements in microscopy, image analysis, molecular cell biology and other cell biological techniques have uncovered key aspects of the cell biological processes like cell polarity, mitotic spindle and cleavage plane orientation, cell cycle length, dynamics of junctional proteins, delamination etc., that occur at different developmental time points and ultimately lead to an expansion of the neocortex. Several new players and the molecular details of how their networking regulates these processes have also been identified. In this review, we will first discuss the diversity of the stem and progenitor cells that are found in the developing neocortex across different mammalian species. We will then proceed to specifically illustrate the cell biological features that are associated with these different stem and progenitor cells, and how these features influence the proliferation, cell fate, morphology and migration of these cells (Figure 1).

## 2 Neocortical Development and Progenitor Cell Types in Developing Neocortex

With the onset of neurogenesis, the neuroepithelial cells (NECs) differentiate into a glial cell population, referred to as apical radial glial cells (aRGCs, also referred to as ventricular radial glia), which give rise to other glial and non-glial progenitor cell types that eventually generate all the neocortical projection neurons. Specifically, the various progenitor cells in the developing neocortex reside in two germinal zones—i) the ventricular zone (VZ), the primary germinal zone; and ii) the subventricular zone (SVZ), a secondary germinal zone. In species with an expanded neocortex, and especially in gyrencephalic species, the SVZ gets further subdivided into an inner SVZ (ISVZ) and an outer SVZ (OSVZ) (Smart et al., 2002), with the OSVZ becoming the most prominent proliferative zone in these species (Smart et al., 2002; Fietz et al., 2010; Hansen et al., 2010; Borrell and Reillo, 2012). An OSVZ-like zone has also been reported in the lissencephalic mouse and rat neocortex at later stages of embryonic neurogenesis (Martínez-Cerdeño, 2012; Vaid et al., 2018). Within these germinal zones, based on the location of the nucleus at mitosis, the progenitor cells can broadly be divided into two principal classes, i) apical progenitors (APs), which undergo mitosis at the ventricular surface of the VZ (Figure 1); and ii) basal progenitors (BPs), which undergo mitosis in the SVZ (Haubensak et al., 2004; Miyata et al., 2004; Noctor et al., 2004; Fietz et al., 2010; Hansen et al., 2010; Shitamukai et al., 2011; Wang et al., 2011; Betizeau et al., 2013).

At the cell biological level, APs (i.e., NECs, aRGCs) remain integrated into the apical adherens junction (AJ) belt throughout their cell cycle, their nucleus undergoes apical-to-basal and basal-to-apical migration in concert with the cell cycle (interkinetic nuclear migration, INM), and their mitosis at the ventricular surface reflects the presence of an apical primary cilium throughout interphase. aRGCs retain their basal process at mitosis. This is similar to mouse E10.5 NECs (Kosodo et al., 2008) but in contrast to early human NECs, which have been reported to retract the basal process at mitosis (Subramanian et al., 2017). Furthermore, a subtype of APs called short neural precursors (SNPs) or apical intermediate progenitor cells (aIPCs) have been identified in developing mouse neocortex that retract their basal process at mitosis such that it remains as a small truncated process within the VZ (Gal et al., 2006). Recently, aRGCs have also been reported, during mid-neurogenesis in the developing human neocortex, to exist as a subtype with a truncated basal process; however, unlike mouse SNPs/aIPs, the basal process of human truncated aRGCs terminates in the OSVZ (Nowakowski et al., 2016). Perhaps just a coincidence, but it is interesting to note that in both mouse and human, SNPs/aIPs and aRGCs with a truncated basal process, respectively, appear around the time when about 1/3 of neurogenesis is completed (E12.5 in mouse and GW16.5 in human) (Gal et al., 2006; Nowakowski et al., 2016). Furthermore, regarding the truncated aRGCs, these progenitor cells can provide a scaffold for newborn BPs to ensure that the latter progenitor cells reach, and seed, the OSVZ. In addition to these various types of APs, the developing dorsal telencephalon of gyrencephalic species has been reported to also harbor yet another cell type called subapical progenitors, where the cells are integrated into the AJ belt and maintain a basal process that contacts the basal lamina (like conventional aRGCs), but undergo mitosis in the VZ at a position basal to the ventricular surface (Pilz et al., 2013).

BPs originate in the VZ by divisions of aRGCs. The newly generated BP retracts its apical process from the ventricular surface and AJ belt and moves to the SVZ (Figure 1 please see *Delamination* below). BPs are further divided into two main types—basal intermediate progenitor cells (bIPCs) and basal radial glial cells (bRGCs, also referred to as outer radial glia). bIPCs are multipolar cells and are the prominent BP type in mouse (Miyata et al., 2001; Haubensak et al., 2004; Noctor et al., 2004), where they have limited proliferative capacity (see below for a definition of this term) and usually undergo only 1-2 rounds of symmetric divisions for their amplification in the SVZ before undergoing symmetric consumptive division to generate neurons (Noctor et al., 2004).

In contrast, bRGCs are the prominent BP type in species with an expanded neocortex (Lukaszewicz et al., 2005; Fietz et al., 2010; Hansen et al., 2010; Reillo et al., 2011; Kelava et al., 2012; Betizeau et al., 2013; Lamonica et al., 2013), but are rare in lissencephalic species like mouse (Shitamukai et al., 2011; Wang et al., 2011). Interestingly, a recent study demonstrated an abundance of bRGC as high as that is found in gyrencephalic species in the developing mouse medial neocortex towards the end of neurogenesis (Vaid et al., 2018). At the cell biological level, bRGCs are characterized by radial processes. They typically extend a basal process (maintained at mitosis) that may contact the basal lamina; in addition, they may extend an apically directed process that, however, lacks contact with the ventricle (Lukaszewicz et al., 2005; Fietz et al., 2010; Hansen et al., 2010; Borrell and Reillo, 2012; Betizeau et al., 2013; Kalebic et al., 2019). bRGCs have high proliferative capacity. We define the term “proliferative capacity” as the ability of a given neural progenitor type to undergo multiple rounds of either symmetric proliferative or asymmetric self-renewing divisions, which results in an increased number of daughter cells. For example, about 40% of bRGCs in developing macaque neocortex have been shown to undergo symmetric proliferative divisions, generating up to six daughter cells per bRGC (Betizeau et al., 2013). An increase in the relative population of bRGCs has been shown to induce/increase cortical folding (Stahl et al., 2013).

## 3 Cell Biological Aspects of the Progenitor Cell Types in Developing Neocortex

### 3.1 Apical Plasma Membrane and Basal Process

#### 3.1.1 Apical Domain

The apical-most surface of the NECs and aRGCs that directly faces the ventricles constitutes the apical domain of the plasma membrane of these cells. This domain can be visualized as a cadherin–negative, prominin-1–positive segment of the plasma membrane (Kosodo et al., 2004). Despite being a small area (Figure 1) (corresponding to only 1–2% of the total plasma membrane), the apical plasma membrane provides crucial polarity cues that influence the cell fate of the dividing cell (Please see below the sections on *Primary cilium and centrosomes*, *Adherens junctions* and *Delamination*) and serves as a docking site for several signaling ligands through their receptors that are expressed on its surface facing the ventricular lumen (Taverna et al., 2014). Symmetric proliferative divisions of NECs prior to neurogenesis and of aRGCs during neurogenesis typically exhibit a vertical cleavage plane, which results in an equal distribution of the apical membrane to the two daughter cells.

In contrast, an oblique or even horizontal cleavage plane during neurogenesis that bypasses the AJ belt, which would result in the distribution of the apical membrane to only one of the daughter cells, predicts an asymmetric, self-renewing plus BP-genic aRGC division (Kosodo et al., 2004; Noctor et al., 2008; Kawaue et al., 2019). Fate-wise asymmetric aRGC division can also occur when the cleavage plane does not bypass, but bisects—albeit not necessarily equally—the apical domain (Figure 1). In such asymmetric divisions of aRGCs, the two daughter cells may inherit size-wise nearly equal portions of the apical domain, that however are unequal with regard to the fate determination of the two daughter cells . Specifically, it has been proposed that the asymmetric inheritance of a small sub-domain of the apical plasma membrane may be linked to a proliferative vs. neurogenic fate of the daughter cells. To address this issue, Shitamukai et al. (2011) visualized the inheritance of the apical domain using ZO-1-EGFP and PAR3-EGFP, both of which in epithelial cells are known to be localized also to the AJs (Itoh et al., 1993; Takekuni et al., 2003). Therefore, the readout of apical domain inheritance in the Shitamukai et al. (2011) study included a significantly larger area than just the apical plasma membrane. In contrast, Kosodo et al. (2004) used the cadherin-negative segment of the apical domain as a readout and showed that the inheritance of this very small portion of the apical domain correlated with the asymmetric divisions of aRGCs.

In extreme, rare cases, however, when the cleavage plane is parallel to the ventricular surface, the apical daughter cell inheriting the complete apical domain, and no basal domain, becomes postmitotic (Shitamukai et al., 2011). These latter results indicate that the inheritance the apical domain alone is not sufficient for the daughter cell to retain aRGC fate (please see below for the role of basal process in cell fate and proliferation capacity).

#### 3.1.2 Basal Domain

The basolateral membrane accounts for the major fraction of the plasma membrane of NECs, aRGCs and bRGCs. On its basal-most end, a structure called the basal endfoot makes direct contact with the basal lamina in the case of NECs and canonical aRGCs, and may do so in the case of bRGCs (Haubst et al., 2006; Taverna et al., 2014). The basal lamina is a sheet of extracellular matrix (ECM) composed mainly of type IV collagen, nidogen, members of the laminin family and heparan sulphate proteoglycans, such as perlecan and agrin (Erickson and Couchman, 2000), and is enriched with a variety of growth factors (Kazanis and Ffrench-Constant, 2011; Wade et al., 2014). The basal endfoot contacting the basal lamina is a highly dynamic structure (Yokota et al., 2010) that can transduce signals from the ECM-rich basal lamina (Jeong et al., 2013; Singer et al., 2013). The basal endfoot has also been shown to spatially restrict several mRNAs and RNA binding proteins, which may be involved in transducing pro-proliferative signals (Tsunekawa et al., 2012; Pilaz et al., 2016).

#### 3.1.3 Basal Process

Concomitant with the transition of NECs to aRGCs, the initally cuboidal NECs become more elongated and, keeping pace with the increasing cortical wall thickness, their basal-most segment, referred to as the basal process, becomes very thin and grows in length, spanning the neuronal layers to reach the basal lamina (Taverna et al., 2014). Most RGCs (both aRGCs and bRGCs) retain their basal process during mitosis (Miyata et al., 2001; Noctor et al., 2001; Fish et al., 2006; Fietz et al., 2010; Betizeau et al., 2013), and only a subset retracts it at mitosis (Gertz et al., 2014). These data suggest that from the onset of neurogenesis onwards, basal process retention through mitosis serves some important function. Originally being thought to serve primarily as a scaffold for neurons and other cells to migrate on (Rakic, 1972; Noctor et al., 2001; Noctor et al., 2004; Silva et al., 2019), the basal process has now emerged, in addition, as an active subcellular compartment involved in signaling and cell fate specification and especially as a key cell biological feature conferring high proliferative capacity to the bRGCs leading to the evolutionary expansion, and likely the gyrification, of the neocortex (Uzquiano et al., 2018; Kalebic and Huttner, 2020), discussed below in more detail).

Regarding the basal process of aRGC, live-imaging experiments in mouse have shown that the basal process is asymmetrically inherited during mitosis (Miyata et al., 2001) and that the daughter cell inheriting the basal process usually maintains an aRGC cell fate (Konno et al., 2008; Lamonica et al., 2013). In addition, for both aRGC and the bRGC divisions, the daughter cell that does not inherit the basal process can regrow it after division (Miyata et al., 2001; Betizeau et al., 2013), and active Notch signaling has been shown to induce this regrowth (Shitamukai et al., 2011). These results support the notion that the inheritance of the basal process is not necessary to remain an aRGC or bRGC. For future research, it will be important to investigate if additional mechanisms exist that underlie the regrowth of a basal process.

#### 3.1.4 Basal Process Branching

The basal process may show several small branches along its length (Kalebic et al., 2019). In addition to serving as a scaffold for migrating projection neurons, the long primary basal process and its branches allow the interaction with the surrounding ECM and various other cell types, e.g., with interneurons and blood vessels. This adds to the diversity of signals that the progenitor cells bearing such long basal processes can experience, and likely to their increased proliferative capacity. An inter-species comparison of BP morphology has shown that the branching index of the processes in BPs (the total number of all processes divided by the number of primary processes) increases from mouse to ferret to human (Kalebic et al., 2019). Furthermore, it was shown that the paralemmin family member PALMDELPHIN (PALMD), *via* integrin signaling, promotes the process growth of BPs, and this increase in process number and branching index is directly related to their proliferative capacity (Kalebic et al., 2019). These findings establish a strong role of increased surface area in the proliferative capacity of BPs.

Among the bRGCs, in addition to an increase in the overall branching index of the basal process, the basal process has been shown to display diversity in its morphology. Specifically, in addition to the previously described morphotypes (Betizeau et al., 2013), new morphotypes with 2 basal processes were identified specifically in gyrencephalic species (Kalebic et al., 2019). These bifurcated basal processes have been shown split either nearby the cell body or away from the cell body. These new morphotypes are particularly interesting in light of the notion that the basal process is a key feature of highly proliferative bRGCs and therefore a crucial element in cortical evolution (Smart et al., 2002; Fietz et al., 2010; Hansen et al., 2010; Reillo et al., 2011; Betizeau et al., 2013; Lamonica et al., 2013; Kalebic et al., 2019). Kalebic et al. (2019) also showed that PALMD can increase the basal process number of bRGCs in gyrencephalic species but not in lissencephalic species. This is an interesting finding because it suggests an evolutionary difference in the basal process-generating molecular machinery between gyrencephalic and lissencephalic species. An interesting line of future research will be to compare the proliferative capacity of these different bRGC morphotypes and link it to the corresponding morphology. Along this line, bRGCs with both basal and apically directed processes have been shown to have a higher proliferative capacity than bRGCs with either an apically directed or a basal process only (Betizeau et al., 2013).

#### 3.1.5 Basal Process Splitting

During cell division the basal process of mouse E10.5 NECs has been shown to get split before anaphase onset and to then be inherited either symmetrically or asymmetrically between the two daughter cells (Kosodo et al., 2008). As this basal process splitting during NEC division involves anillin and the cytokinesis machinery, it is unlikely to be mechanistically related to the basal process branching of bRGCs discussed above.

#### 3.1.6 Mitotic Somal Translocation

The basal process also plays role in another cell biological event associated specifically with bRGCs—mitotic somal translocation (MST), an actin-myosin–driven fast translocation of the nucleus along the radial fiber before cytokinesis (Hansen et al., 2010; Betizeau et al., 2013; Gertz et al., 2014; Ostrem et al., 2014). MST has been proposed to play a role in the evolutionary expansion of the neocortex because the frequency of bRGCs undergoing MST and the frequency a pial-directed trajectory (which can likely expand the OSVZ) has been shown to increase from ferret to macaque to human (Betizeau et al., 2013; Gertz et al., 2014; Ostrem et al., 2014).

#### 3.1.7 Fanning of Basal Processes

In its role as the scaffold for the migrating neurons, the basal process of the bRGCs has further gained an evolutionary importance as it has been shown that during the generation of the supragranular layers in primates, the aRGC basal process no longer contacts the pial surface (referred to as truncated aRGC). Rather the aRGC basal process instead terminates in the OSVZ [(Nowakowski et al., 2016) and the references therein], and the neurons destined for the supragranular layers therefore migrate along the bRGC basal process (Nowakowski et al., 2016). An additional evolutionary feature related to the bRGC basal processes that can directly influence the gyrification in the developing neocortex is the observation that these basal processes have been shown to fan out during development, and this fanning has been shown to be necessary to promote the tangential dispersion of the migrating neurons, which allows a significant growth in the surface area of the developing neocortex (Reillo et al., 2011; Lewitus et al., 2013).

### 3.2 Mitotic Spindle and Cleavage Plane Orientation

As mentioned earlier, aRGCs, like NECs, are polarized cells, and their apical-basal polarity is critical to the cell fate of their daughter cells. The cleavage plane orientation upon cell division determines how the cellular components, especially the polarity-related ones, will be distributed between the two daughter cells. The cleavage plane orientation is determined by the orientation of the mitotic spindle. It is therefore not surprising that a premature neuronal differentiation and cortical disorders such as lissencephaly or microcephaly are associated with mutations in genes that have a role in mitotic spindle orientation or mitotic spindle organization (Feng and Walsh, 2004; Fish et al., 2006; Gauthier-Fisher et al., 2009; Garcez et al., 2015).

In developing mouse neocortex, symmetric proliferative divisions of NEC have been shown to exhibit a vertical cleavage plane, that is, parallel to their apical-basal axis, distributing the cellular components equally between the two daughter cells (Kosodo et al., 2004). With the onset of cortical neurogenesis and its progression, the cleavage plane orientation of aRGCs may be either vertical or oblique, with the frequency of oblique cleavage plane orientation increasing with the progression of neurogenesis (Figure 1) (Haydar et al., 2003; Konno et al., 2008; Wang et al., 2009; Asami et al., 2011; Shitamukai et al., 2011). In the developing mouse neocortex, such oblique aRGC divisions have been shown to generate BPs, both bIPs and bRGCs (Wang et al., 2009; Asami et al., 2011; Shitamukai et al., 2011; Wong et al., 2015). Interestingly, oblique or even horizontal orientations of the aRGC cleavage plane can be associated with the generation of bRGCs also in gyrencephalic species (Shitamukai et al., 2011; Lamonica et al., 2013; Pilz et al., 2013; Gertz et al., 2014). In line with the much higher proportion, among the BPs, of bRGCs in human than mouse, the frequency of such oblique and horizontal aRGC cleavage plane orientations is significantly higher in humans than in rodents (Lamonica et al., 2013; Pilz et al., 2013). Additionally, loss of function mutations causing spindle randomization have been shown to cause an increase in the generation of bRGCs in embryonic mouse neocortex (Shitamukai et al., 2011). These results raise the possibility that a downregulation of the machinery ensuring a horizontal mitotic spindle, and hence a vertical cleavage plane orientation may have contributed to neocortex expansion during evolution.

In this context, it has previously been demonstrated that an LGN-dependent decrease specifically in the astral microtubules reaching the basal or the apical region of the cell cortex (especially the basal region) triggers a change from vertical to oblique spindle orientation, leading to the shift from symmetric to asymmetric aRGC divisions in embryonic mouse neocortex (Figure 1) (Mora-Bermudez et al., 2014).

Another interesting feature associated with the mitotic spindle is its highly dynamic nature during metaphase. The mitotic spindle of APs has been shown to rotate, even making several turns, before it comes to rest just prior to the onset of anaphase (Adams, 1996; Haydar et al., 2003). This implies that the tethering of the astral spindle microtubules to the actin cytoskeleton at the cell cortex is not very strong during most of metaphase. One possible explanation for this spindle rotation could therefore be the active and ongoing rearrangement of the actin configuration at the cell cortex with which the astral microtubules eventually have to establish a strong contact. Another speculative explanation is that the duration of this spindle rotation provides a short plastic period to the dividing cell to allow it to sense its environment for the last time before the division and re-orient the cleavage plane appropriate for the environment at the time of cleavage.

### 3.3 Primary Cilium and Centrosomes

Primary cilia are non-motile cilia. They consist of a microtubule-based cytoskeletal structure surrounded by ciliary membrane, which in epithelial cells like NECs and aRGCs is an extension of the apical plasma membrane. The primary cilium of aRGCs protrudes into the lumen of the ventricle to receive, and transduce, the signals from signaling molecules, such as Wnt and Shh, that are present in the ventricular fluid (Corbit et al., 2005; Eggenschwiler and Anderson, 2007; Rohatgi and Scott, 2007; Gerdes and Katsanis, 2008; Goetz et al., 2009; Lehtinen and Walsh, 2011; Louvi and Grove, 2011; Oberst et al., 2019). In addition to serving as an antenna for such signals, the components of the primary cilium of NECs and aRGCs play essential role in various other cell biological processes like INM, mitotic spindle formation, the mode of cell division, and the stability of the apical AJ belt, which will be discussed below.

In NECs and aRGCs at interphase, the mother centriole of the centrosome (the older one of the two centrioles inherited upon the birth of the cell) constitutes the basal body of the apical primary cilium (Kumar and Reiter, 2021; Wilsch-Bräuninger and Huttner, 2021) and is therefore tethered to the apical plasma membrane (Figure 1). During the cell cycle of NECs and aRGCs, the apical primary cilium is not disassembled, and the mother centriole hence not detached from the apical cell cortex, until early prophase. In other words, the mother centriole remains tethered to the apical plasma membrane until mitosis onset. Moreover, the nucleus of a NEC or aRGC is located at a non-apical position within the VZ during interphase due to apical-to-basal INM. Hence, the mother centriole can only function, as part of a centrosome, as mitotic spindle pole in cell division if the nucleus migrates towards this centrosome for mitosis via basal-to-apical INM. SUN-domain and KASH-domain proteins link the microtubule appendages of the centrosome to the nucleus and transduce the contracting forces from the microtubules to the nucleus during the basal-to-apical migration of the nucleus (Zhang et al., 2009).

What about the second centrosome required to form a proper mitotic spindle? The two centrioles (one of which is the basal body of the apical primary cilium) separate and duplicate during the G1/S phase. The two new pairs of centrioles—the mother centriole with its duplicate and the daughter centriole with its duplicate—then form the two centrosomes required to build a proper mitotic spindle. During late G2/early prophase, the primary cilium gets resorbed by the cell, and the mother centriole switches its role from being the basal body to serve, along with its duplicate, as one of the mitotic spindle poles. From the resorbed components of the primary cilium, the mother centriole retains a large part of its distal and subdistal appendages (Breslow and Holland, 2019; Tischer et al., 2021) and remains associated with a remanent of the ciliary membrane; these three components—mother centriole, associated ciliary membrane remnant, and duplicated centriole—undergo endocytosis prior to this centrosome becoming a mitotic spindle pole (Figure 1) (Paridaen et al., 2013). Following cytokinesis, these additional components associated with the mother centriole accelerate the re-establishment of the—typically apical—primary cilium in the daughter cell inheriting the mother centriole, which allows for a faster responsiveness to stem cell fate-promoting factors in the environment, notably the ventricular fluid (Anderson and Stearns, 2009; Wang et al., 2009; Piotrowska-Nitsche and Caspary, 2012; Paridaen et al., 2013).

In the non-aRGC daughter of an asymmetric aRGC division, which typically is a BP, from the very beginning of neurogenesis, the re-establishment of the primary cilium shows a key cell biological difference when compared to the re-establishment of the apical primary cilium in the aRGC daughter. In these newborn BPs, instead of generating an apical primary cilium, the inherited centrosome generates a basolateral primary cilium, very close (but basal to) to the apical AJ belt (Figure 1) (Wilsch-Bräuninger et al., 2012). This basolateral positioning of the primary cilium is the first observed cell biological indicator of BP delamination, and is likely to prevent this cilium from receiving macromolecular signals from the ventricular lumen, which do not cross the AJ belt. The genetic programs that specifically regulate the basolateral positioning of the primary cilium have not yet been elucidated and therefore remain an open field for future research.

Recent studies have shown an emerging role of centrosome-associated proteins in the delamination of BPs (see below) by regulating the interaction between the cytoskeleton and AJs, which eventually affects the stability of the AJs. For example, in BP-genic APs and newborn BPs, the AT-hook protein AKNA localizes to subdistal appendages on the mother centriole. By influencing the actin re-modeling and AJ stabilization, AKNA regulates the apical constriction and the delamination of the newborn BP (Camargo Ortega et al., 2019). Similar to AKNA, another centriolar protein, Talpid3, which localizes to the distal end of the mother centriole (Yin et al., 2009; Kobayashi et al., 2014; Wang et al., 2020), has been shown to maintain the integrity of the AJ by modulating microtubule stability (Wang et al., 2020).

### 3.4 Adherens Junctions

As mentioned above, aRGCs, the cells that directly or indirectly give rise to all the projection neurons of the neocortex, maintain an apicobasal polarity throughout cortical development. This apicobasal polarity of aRGCs is crucial for proper cortical development, as it has a direct influence on aRGC morphology, architecture of the ventricular surface, aRGC size, mode of aRGC division, and radial BP migration (Chenn and Walsh, 2002; Machon et al., 2003; Woodhead et al., 2006; Stocker and Chenn, 2015; Veeraval et al., 2020). The apical belt of AJs, the cadherin-based cell–cell adhesion complexes, demarcates the border between the lateral and apical plasma membrane and is a key player in maintaining the apicobasal polarity of the aRGCs. This is so because aRGCs lose functional tight junctions during neural tube closure (Aaku-Saraste et al., 1996) and therefore rely solely on the AJ belt to maintain their polarity and tissue architecture. Mutations in key junctional proteins, leading to a failure of AJ assembly, have pleotropic effects, leading to loss of aRGC polarity (Lien et al., 2006; Kadowaki et al., 2007; Kim et al., 2010; Katayama et al., 2011; Cappello et al., 2012; Yamamoto et al., 2013; Gil-Sanz et al., 2014; Taverna et al., 2014; Schmid et al., 2014; O’leary et al., 2017; Rakotomamonjy et al., 2017).

Interactions between polarity proteins and AJ components facilitates AJ assembly. Thus, Lgl1 directly binds to and promotes the internalization of N-cadherin (Jossin et al., 2017). The Par3 protein, which recruits Par6, aPKC and Cdc42 to form the Par3/Par6/aPKC/Cdc42 polarity complex is localized to the apical cell cortex (Manabe et al., 2002; Kosodo et al., 2004; Cappello et al., 2006; Costa et al., 2008). aPKC phosphorylates and deactivates Lgl1 and excludes the Lgl/Dlg/Scribble polarity complex from the apical cell cortex, and therefore this complex gets restricted to the apical-most region of the lateral membrane, promoting internalization of N-cadherin at this lateral membrane domain. aPKC-mediated phosphorylation of Lgl1 also inhibits the N-cadherin-Lgl1 interaction (Jossin et al., 2017), and therefore N-cadherin accumulation and AJ formation gets restricted to the basolateral-apical boundary.

AJs influence well-known cell fate determination signals and *vice versa*. Thus, Notch, a key stem cell determinant, associates with the cadherin complex and is localized to AJs. Conversely, AJ assembly has been shown to be required for Notch activation (Del Bene et al., 2008; Bultje et al., 2009; Ohata et al., 2011; Hatakeyama et al., 2014). Numb, a known inhibitor of Notch signaling (Frise et al., 1996; Spana and Doe, 1996; Rasin et al., 2007), also directly interacts with cadherins, is localized to cadherin-positive recycling endocytic vesicles at AJs, and is required for the maintenance of AJs (Rasin et al., 2007).

Recently, the AJ component Afadin has been shown to have a role in mitotic spindle orientation. Afadin deletion was shown to increase oblique aRGC divisions, which subsequently increased the level of BPs (Rakotomamonjy et al., 2017). Further support for Afadin’s role in mitotic spindle orientation was reported in other epithelial systems (HeLa cells and human colorectal adenocarcinoma cell line Caco-2), where binding of Afadin to F-actin and LGN has been shown to promote symmetric proliferative divisions (Carminati et al., 2016).

### 3.5 Cell Delamination

Delamination is the process by which a cell, typically a newborn BP, loses its apical plasma membrane and its contact with the AJ belt and retracts its apical endfoot. BP delamination is therefore the first step in, and a requirement for, the migration of BPs to the SVZ. Since the generation of BPs has an immense influence on cortical expansion, BP delamination is an extremely important, and—mechanistically and temporally—tightly regulated, cell biological event in the developing neocortex.

Dynamic changes in the microtubule–actin–AJ configuration at the apical endfoot, (constriction of the AJ belt, downregulation of cadherin expression, etc.) are key events associated with delamination, which are mediated by transcriptional suppression of AJ-related components and by other posttranscriptional cascades to regulate cell adhesion and cytoskeletal architecture.

Upon asymmetric aRGC division, depending on the mitotic spindle and hence cleavage plane orientation (please see *mitotic spindle*), the daughter cell destined to delaminate, typically a newborn BP, may be born with or without inherited AJs and with or without apical domain. If a newborn, not yet delaminated BP has inherited AJs, the AJ components are actively suppressed to disassemble the AJs prior to delamination. Loss of cadherin, a crucial component of AJs, has been shown enhance cell delamination, increasing the production of both bIPs and bRGCs (Itoh et al., 2013; Martinez-Martinez et al., 2016). Moreover, the daughter cell inheriting less of the apical membrane and less of the AJ components experiences a downregulation of the Notch signaling, which leads to the stable expression of proneural genes like Ngn2 (Vaid and Huttner, 2020). Ngn2 promotes the expression of insulinoma-associated 1 (Insm1), Scratch 1 and Scratch 2, all members of the SNAG family (Vaid and Huttner, 2020). Insm1 has recently been shown to promote the expression of Robo2, a transmembrane receptor of the ROBO family, and to down-regulate the expression of Plekha7, an AJ belt-specific protein, causing the AJs to disassemble (Farkas et al., 2008; Tavano et al., 2018; Vaid and Huttner, 2020).

If a BP is born with apical plasma membrane, its delamination not only involves getting out of the AJ belt, but also getting rid of apical plasma membrane. This can be achieved either by an abscission of the apical endfoot, where the apical process is constricted in an actomyosin-dependent manner and gets pinched off, or by endocytosis of apical plasma membrane followed by its degradation (Das and Storey, 2014).

As discussed above, centrosome-associated proteins, by modulating microtubule and actin stability, also influence AJ stabilization and therefore regulate delamination (Camargo Ortega et al., 2019; Wang et al., 2020). Recently, the microtubule-associated protein Lzts1 has been shown to inhibit microtubule assembly and to activate the actomyosin system at the apical endfoot of newborn BPs, and hence functions in BP delamination by altering the organization of the apical AJ belt (Kawaue et al., 2019).

### 3.6 Cell Cycle Parameters

BPs generated during neurogenesis in the embryonic mouse neocortex have been shown to have a specific increase in the length of the G1 phase as compared to the aRGCs they are derived from (Calegari et al., 2005; Lange et al., 2009; Arai et al., 2011). In fact, increasing the length of the cell cycle of NECs is sufficient to increase the appearance of neuronally committed progenitors and to induce premature neurogenesis (Calegari and Huttner, 2003). Comparison of the cell cycle parameters of aRGCs undergoing symmetric proliferative divisions vs. aRGCs undergoing asymmetric BP-genic divisions revealed a substantially longer S-phase in the former aRGC subpopulation (Arai et al., 2011). This suggests that aRGCs undergoing divisions to expand their pool size invest more time into the quality control of the replicated DNA.

Not only cell cycle parameters of APs in interphase, but also of APs in mitosis have been observed to differ between proliferating and BP-genic APs. Specifically, it was found that prometaphase plus metaphase is longer in proliferating than BP-genic APs in embryonic mouse neocortex, with the other phases of mitosis (prophase, anaphase and telophase) showing no significant difference between these two AP subpopulations (Mora-Bermudez et al., 2016).

A comparison of M-phase length of APs in embryonic mouse neocortex, chimpanzee cerebral organoids and fetal human neocortex revealed that the length of AP M-phase increases from mouse to chimpanzee to human (Mora-Bermudez et al., 2016). Intriguingly, among the primates, the M-phase length difference reflected the specific lengthening by ≈50% of metaphase in human APs when compared to chimpanzee or orangutan; interestingly, this metaphase lengthening was only observed at an early stage of cortical development (Mora-Bermudez et al., 2016).

Taken together, these cell cycle parameter analyses show that although the BP-genic APs in embryonic mouse neocortex increase their cell cycle length, specifically the length of G1, compared to proliferating APs, they spend significantly less time in S-phase and in prometaphase-metaphase, the phases where quality control of DNA replication and the preparation for accurate chromosome segregation, respectively, take place. These findings imply that with regard to neurogenesis in the developing neocortex, the accuracy/fidelity of these processes is ensured at an early step, when aRGCs expand their pool size *via* symmetric proliferative divisions. Among the hominids, the specific increase in the metaphase length of mitotic APs in human compared to non-human great apes raises the intriguing possibility that the fidelity of chromosome segregation during the expansion phase of APs in the developing neocortex improved during human evolution.

## 4 Concluding Remarks and Future Directions

In this review, we have addressed cell biological features of the neural stem and progenitor cells in the developing neocortex. One focus has been how specific cell biological events regulate progenitor cell divisions and daughter cell fate. The canonical view of mitotic spindle and cleavage plane orientation being key determinants of aRGC daughter cell fate has evolved in light of studies showing that the relationship between mitotic spindle and cleavage orientation on the one hand and symmetric vs. asymmetric inheritance of apical and basal structures and daughter cell fate on the other hand is more complex than previously thought.

It is now well established that bRGCs, like the other type of BP, the bIPs, originate from aRGCs. However, in contrast to aRGCs, bRGCs show a high diversity in their morphotypes, which impacts their proliferative capacity (Kalebic et al., 2019). Since bRGCs have a key role in the evolutionary expansion of the neocortex, an understanding of the mechanism(s) underlying the generation of this high morphological diversity is very important. Understanding how these bRGC morphotypes evolved requires a more refined investigation of the dynamics of the radial processes in bRGCs and compare them to those in aRGCs.

Lastly, among the features that impact the expansion phase of aRGCs, changes in cell cycle parameters, specifically in the length of S-phase and of metaphase, are emerging as important determinants. This suggests that the underlying genomic changes allowing a tighter control over the quality of DNA replication and the fidelity of chromosome segregation provided advantages for neocortex expansion in the course of primate evolution.
